# A New Contribution on the Sea Slug (Gastropoda: Heterobranchia) Fauna of the Ustica Island Marine Protected Area (Lower Tyrrhenian Sea, Mediterranean)

**DOI:** 10.3390/biology15080647

**Published:** 2026-04-20

**Authors:** Andrea Lombardo, Giuliana Marletta, Renato Chemello, Manuel Ballesteros

**Affiliations:** 1 Independent Researcher, Via Dante 21, 95028 Valverde, Italy; andylombardo94@gmail.com; 2 Independent Researcher, Via Dottor Consoli 57, 95124 Catania, Italy; 3 Department of Earth and Marine Sciences, University of Palermo, Via Archirafi n. 20, 90129 Palermo, Italy; renato.chemello@unipa.it; 4 Departament de Biologia Evolutiva, Ecologia i Ciències Ambientals, Facultat de Biologia, Universitat de Barcelona, Avda. Diagonal, 643, 08028 Barcelona, Spain; mballesteros@ub.edu

**Keywords:** biodiversity, Opisthobranchia, photographic capture technique, sea slugs, Sicily

## Abstract

Ustica is a Sicilian island for which most of the information available on the informal group of sea slugs comes from old and non-specific studies. Consequently, the aim of this study is to provide an updated list of the sea slugs of the Ustica Island Marine Protected Area (MPA). This study, carried out using the “photographic capture technique” in two surveys (early autumn and late spring), led to the finding of 32 species and 14 families of sea slugs. Overall, considering both the literature and current data, a total of 77 species and 33 families of sea slugs have been documented on this island. While these numbers might indicate high species richness compared to other previously investigated Sicilian islands, they might also reflect the fact that Ustica Island is the only one for which numerous malacological studies have been performed.

## Simple Summary

The term “sea slug” refers to an unofficial group of marine creatures characterised by striking body shapes and particular adaptations in their lifestyles. These animals have become well known to the public thanks to their presumed rarity and the spread of underwater photography. In this study, the authors examined the sea slug fauna currently present along the seabed and coastline of Ustica, an island for which most of the information on these animals comes exclusively from historical and old data. Data collection consisted of two campaigns (early autumn and late spring) during which information was gathered through photographs taken during scuba diving and snorkelling sessions. This data collection resulted in the finding of 32 species of sea slugs. Considering these data, together with those found in the literature, a total of 77 species of sea slugs have been found in Ustica. Although this number of species is the highest found to date for a Sicilian island, it is probably due exclusively to the fact that Ustica has been the most-studied Sicilian island over time.

## 1. Introduction

Marine protected areas (MPAs) are delimited areas with the principal goal of protecting and managing marine biodiversity. MPAs usually host increased habitat heterogeneity, an abundance of endemic and threatened species, and a wide range of genotypes [1]. For these reasons, these areas play a crucial role, acting as pivotal refuge areas for biodiversity conservation [2].

In Italy, the first established marine protected areas (MPAs) were Ustica Island and Miramare in 1986 [3]. Ustica is most likely the Sicilian island for which the longest historical series of data on the marine malacofauna is available. The first and most comprehensive study focused on the marine molluscs of Ustica Island was carried out by Chemello [4] between the spring of 1982 and summer of 1984, examining exclusively the marine gastropod fauna in seven sites around the island. Subsequently, through two different data collection campaigns (one carried out in June 1996 and the other one in June 1997), three studies were conducted on the faunas of various molluscan groups, examining three different sites on the island [5,6,7]. Finally, the most recent study on the marine molluscs of Ustica Island was carried out by Castriota et al. [8], who examined the malacofauna present in the maerl beds of the island’s southern coast.

Through the aforementioned studies, a total of 258 species of marine molluscs have been recorded along the coasts of Ustica Island, which corresponds to approximately 19% of the total known malacofauna of the Mediterranean. These data, together with those documented for other marine taxa, suggest that Ustica represents a real hotspot of biodiversity within the Mediterranean Sea [3], and it is also considered one of the most popular destinations for underwater tourism in the Mediterranean [9].

Nowadays, one of the groups of marine invertebrates that is most sought after by divers (especially underwater photographers) is represented by an informal complex of 13 taxa (i.e., Rhodopoidea, Acteonoidea, Ringiculimorpha, Pleurobranchida, Nudibranchia, Doridida, Cephalaspidea, Runcinida, Aplysiida, Pteropoda, Umbraculida, Sacoglossa, and Acochlidiimorpha) of gastropod molluscs belonging to the subclass Heterobranchia. These molluscs, today commonly called sea slugs (formerly known as opisthobranchs), owe their significance to their striking body shapes/colours and their peculiar biological adaptations. These characteristics, together with their presumed and well-known elusiveness, have rendered sea slugs one of the most famous groups of marine organisms among the public [10,11,12,13].

At the Ustica MPA, 62 species of sea slugs have been historically reported along the coastline and seabeds of this island [10] (Table 1). In particular, the faunal data available for Ustica on this informal group of gastropod molluscs are derived almost entirely from non-specific studies [4,5,6,7,8]. Furthermore, the rare specific existing data concern only sporadic reports of single species on the island [14,15,16,17,18], which, in only one case [19], provide additional information beyond the single finding. Moreover, it can be noted that the most recent faunal data on the island’s sea slugs are derived essentially from studies carried out 20–25 years ago [6,7,8]. Consequently, no recent data on the island’s sea slug fauna are currently available [10].

Given Ustica Island’s role as a hotspot of marine biodiversity within the Mediterranean basin [3] and its historical key function as an MPA, this study aimed to produce an updated list of the sea slugs present in this area.

## 2. Materials and Methods

**Figure 1:**
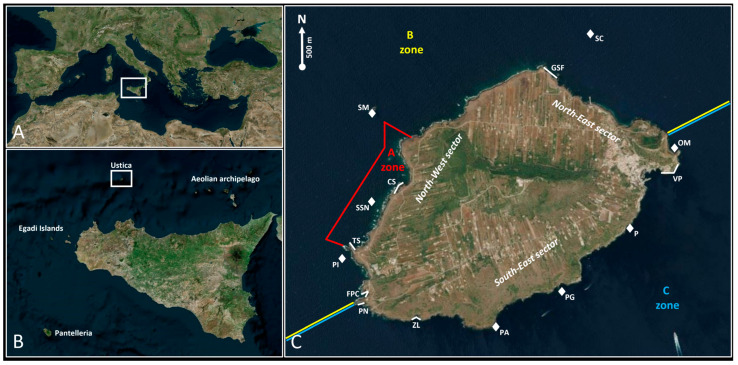
(**A**) Position of Sicily in the Mediterranean Sea; (**B**) position of Ustica in relation to Sicily; (**C**) satellite-based map of Ustica with examined dive sites (represented by white rhombs) and coastal stretches (represented by white lines). The coloured lines indicate the three different MPA zones (blue: C zone; yellow: B zone; red: A zone). The location abbreviations are indicated in Table 2. The maps were created using the free and open-source QGIS.

Ustica is a small island located in the Tyrrhenian Sea, about 67 km off the coast of Palermo (Figure 1A,B). This island represents the summit of a huge inactive volcanic complex that extends for approximately 30 km along the seabed [20,21]. The name Ustica is derived from the Latin word “ustum”, which means “burnt/charred”, a term by which ancient observers described the island’s landscape, mainly characterised by black effusive rocks [22,23].

The island presents a general elliptical shape and a coastal extension of approximately 12 km [22]. In general, almost the entire island’s coastline is characterised by steep cliffs [3]. Specifically, the north and north-east coasts of the island (called “Tramontana” and “Falconiera/Homo Morto”, respectively) present high vertical cliffs characterised by significant rockfalls, while the south coast (called “Mezzogiorno”), having no obvious cliffs (not as significant as the previous ones), is mainly characterised by the presence of numerous caves. Meanwhile, the west coast (called “Ponente” or “Spalmatore”) is lower, being much less steep and jagged compared to the previous ones [3,22].

Through the present study, several localities of Ustica were examined in two different periods, from 27 September 2024 to 1 October 2024 (early autumn—T1) and from 10 June 2025 to 14 June 2025 (late spring—T2).

Data were collected through the use of the photographic capture technique [24,25,26,27]: during scuba dives and snorkelling swims, all observed sea slug specimens (and their egg masses, if present) were photographed through the use of an Olympus TG-6 underwater camera (1/2,3”—12 megapixel) and their depths registered with a SUUNTO D6i underwater computer. The photographs were subsequently examined on a computer to identify the species, count the specimens found, and characterise the substrate types where they were found. Species identification was achieved using the pertinent literature [11,28,29,30,31,32,33] and websites [34,35].

Regarding the morphological identification process, the methodology used followed a systematic hierarchical approach. First, the general body plan of each encountered specimen was assessed to determine its higher taxonomic rank (superorder/order/superfamily). Subsequently, specific diagnostic characteristics (e.g., rhinophores, oral tentacles, parapodia, notum, cerata, and shell presence/structure) and colour patterns were examined to define lower ranks (family/genus/species). The identification of egg masses at the family or genus level was conducted by observing the egg masses’ shapes and colours.

Eight dive sites (Figure 1C) (Table 2) were examined through morning scuba dives (lasting 45–60 min each) carried out following the seabed morphology and examining all habitats/environments where sea slugs could be found. Seven coastal stretches (Figure 1C) (Table 2) were examined through morning snorkelling activities (lasting 45–60 min each) performed following the coastal configuration; also, in this case, all habitats/environments suitable for sea slugs were surveyed. Unfortunately, due to the marine weather conditions, it was not possible to survey all dive sites and coastal stretches in both seasons (Table 2).

**Figure 2:**
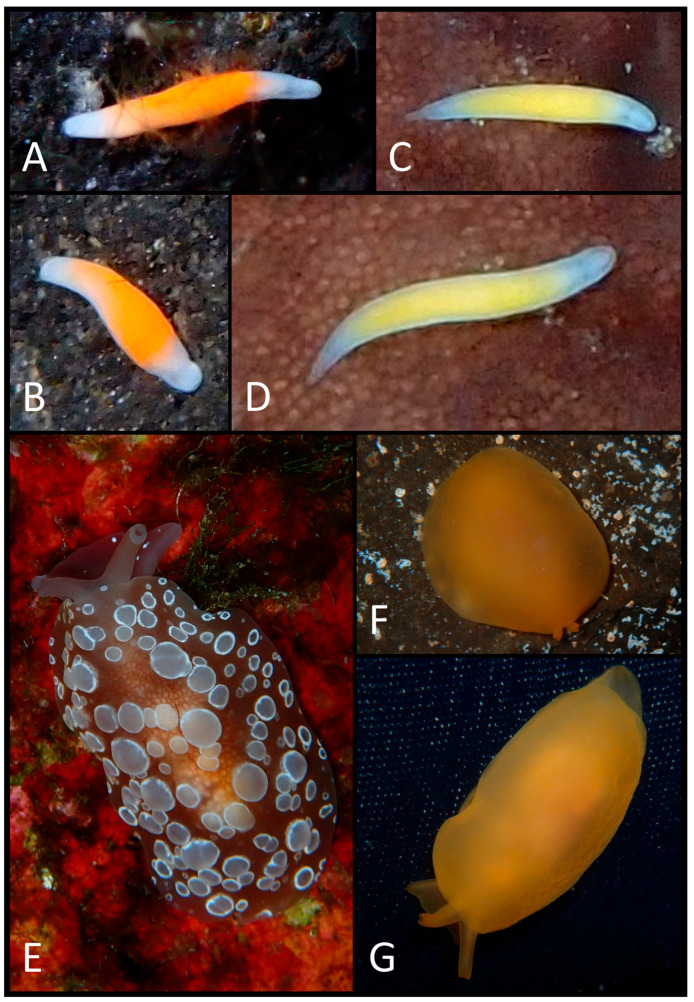
(**A**) Dorsal view of *Rhodope* cf. *salviniplaweni*; (**B**) right dorsal–lateral view of the same specimen; (**C**) dorsal view of *Rhodope* sp. 4; (**D**) the same individual slightly more elongated; (**E**) dorsal view of *Berthella ocellata*; (**F**) a slightly contracted individual of *Berthellina* cf. *edwardsii*; (**G**) the same specimen in elongation.

**Figure 3:**
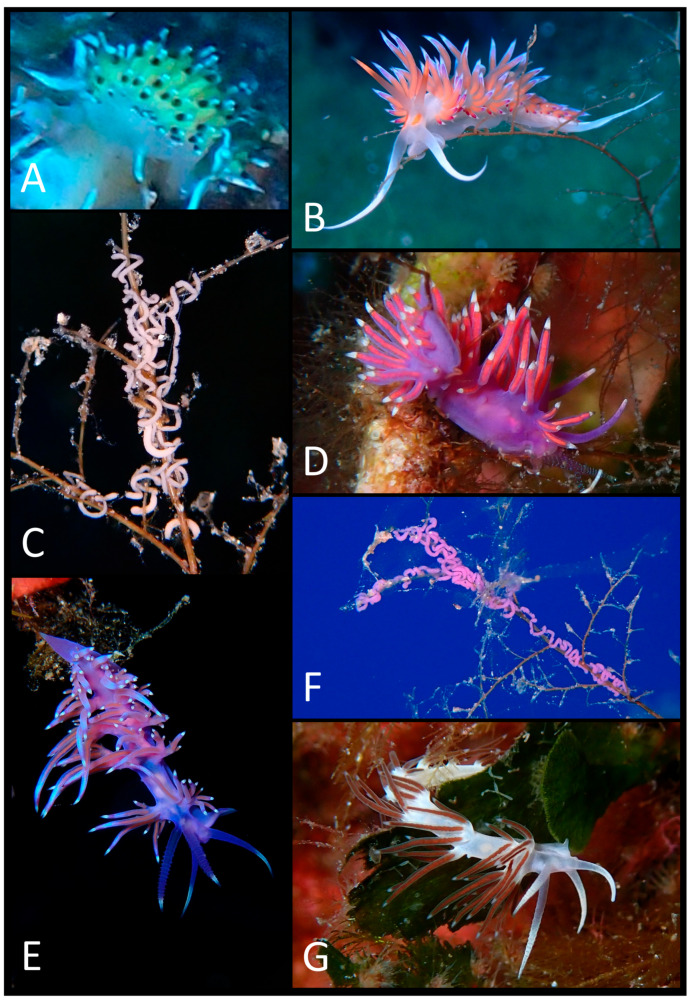
(**A**) *Caloria* cf. *elegans*; (**B**) *Cratena peregrina*; (**C**) *C. peregrina*’s egg mass; (**D**) *Edmundsella pedata*; (**E**) *Flabellina affinis*; (**F**) *F. affinis*’s egg mass; (**G**) *Paraflabellina gabinierei*.

**Figure 4:**
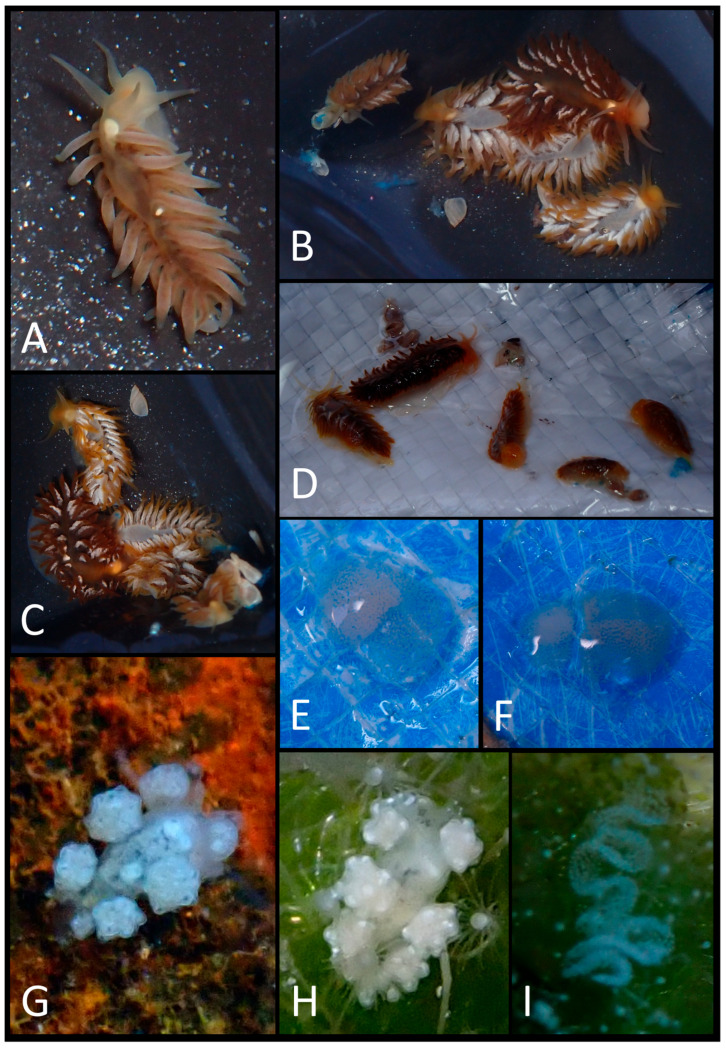
(**A**) *Fiona pinnata*; (**B**) a group of *F. pinnata*; (**C**) the same in a different view; (**D**) the same on their substrata (outside water); (**E**) *F. pinnata*’s egg mass; (**F**) another *F. pinnata*’s egg mass; (**G**) *Doto* sp. 4; (**H**) *Doto* sp. 5; (**I**) *Doto* sp. 5’s egg mass.

**Figure 5:**
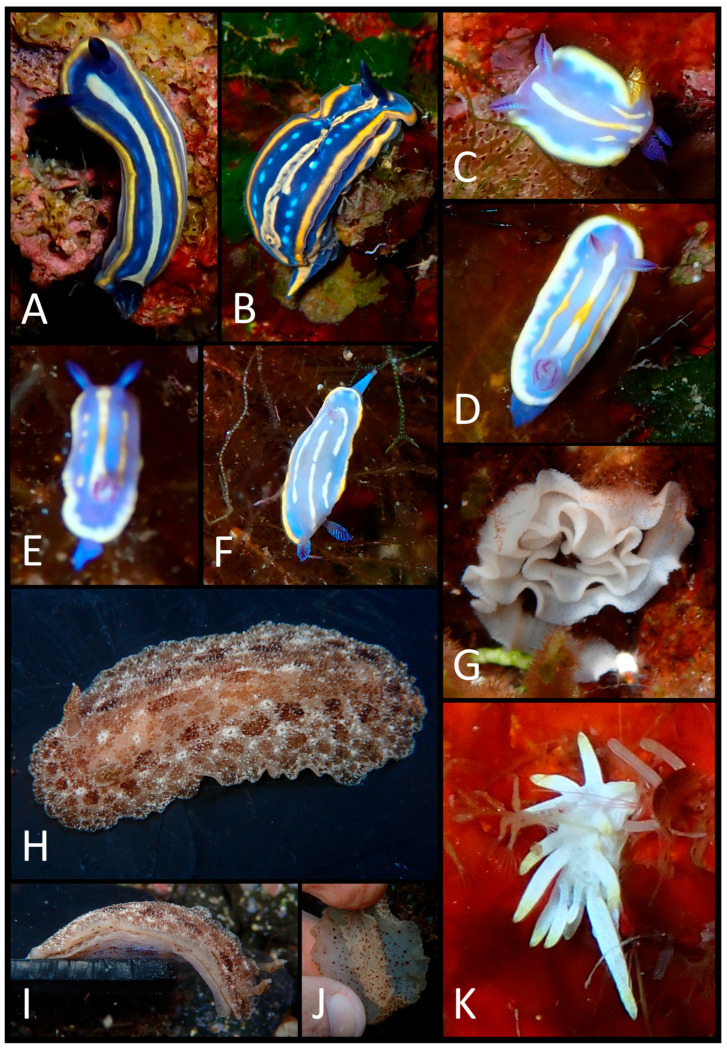
(**A**) Dorsal view of *Felimare tricolor*; (**B**) a damaged individual of *F. tricolor*; (**C**) anterior–dorsal view of *Felimida binza*; (**D**) dorsal view of *F. binza*; (**E**) posterior–dorsal view of *F. binza*; (**F**) *Rudmania krohni*; (**G**) egg mass of *Discodorididae* sp.; (**H**) *Tayuva maculosa*; (**I**) right lateral view of the same individual; (**J**) ventral view of the same; (**K**) *Goniodoridella picohenries*.

**Figure 6:**
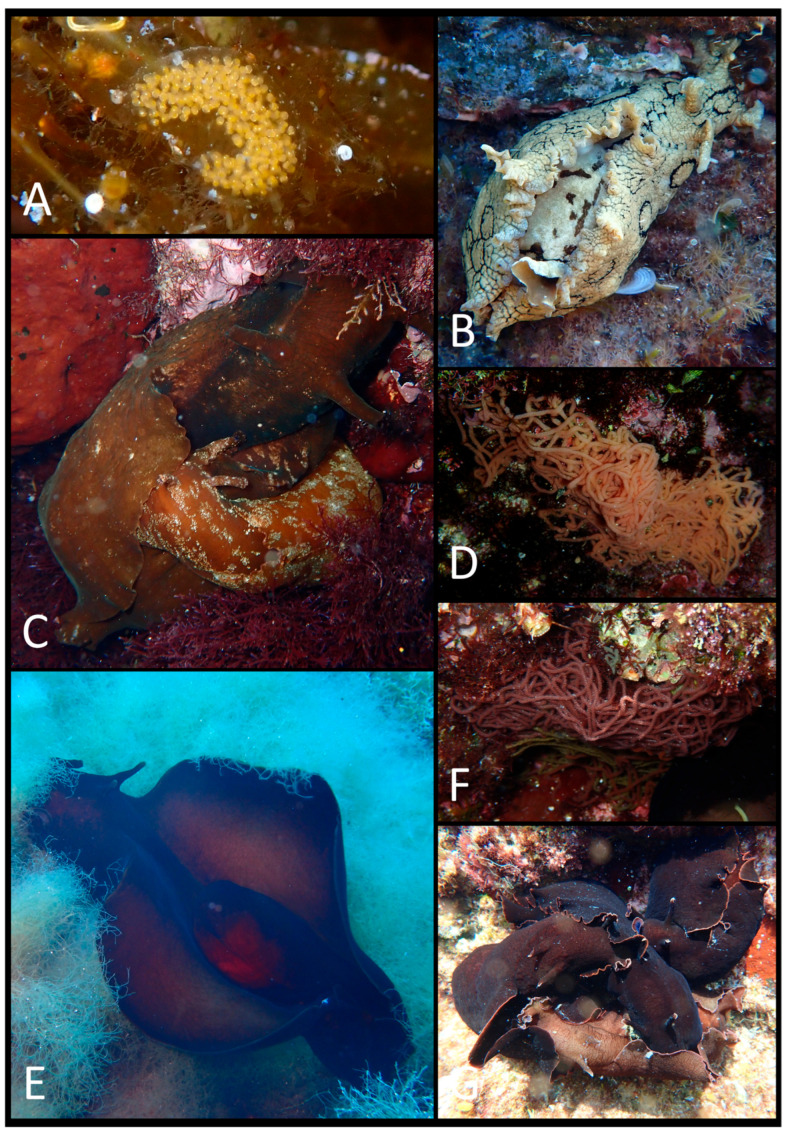
(**A**) *Haloa* sp.’s egg mass; (**B**) *Aplysia dactylomela*; (**C**) two specimens of *A. depilans* during mating; (**D**) *A. depilans*’ egg mass; (**E**) *Aplysia fasciata*; (**F**) *A. fasciata*’s egg mass; (**G**) a group of *A. fasciata* during mating.

**Figure 7:**
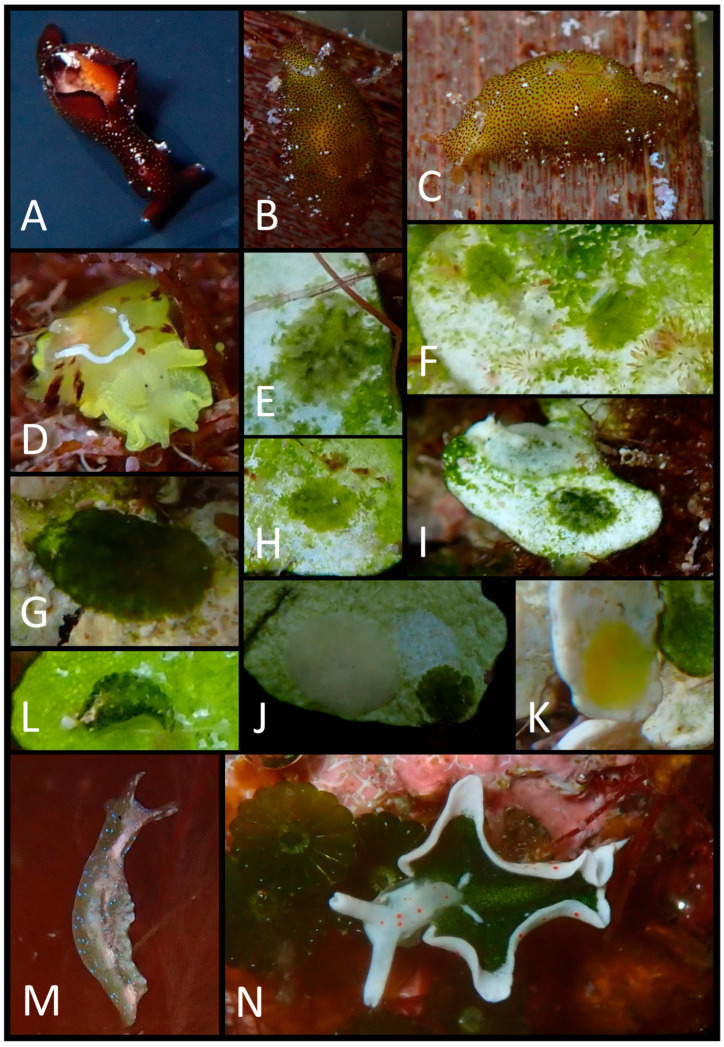
(**A**) A small *Aplysia punctata* specimen; (**B**) left dorsal–lateral view of *Petalifera petalifera*; (**C**) left lateral view of the same individual; (**D**) *Tylodina perversa*; (**E**) dorsal view of a small *Bosellia mimetica*; (**F**) two small *B. mimetica* individuals; (**G**) a medium–large *B. mimetica*; (**H**) a small *B. mimetica*; (**I**) white and green–white *B. mimetica* specimens; (**J**) a *B. mimetica* with a grey egg mass; (**K**) a *B. mimetica*’s yellow egg mass; (**L**) an *Elysia gordanae* juvenile; (**M**) an *E. gordanae* adult; (**N**) *E. timida*.

**Figure 8:**
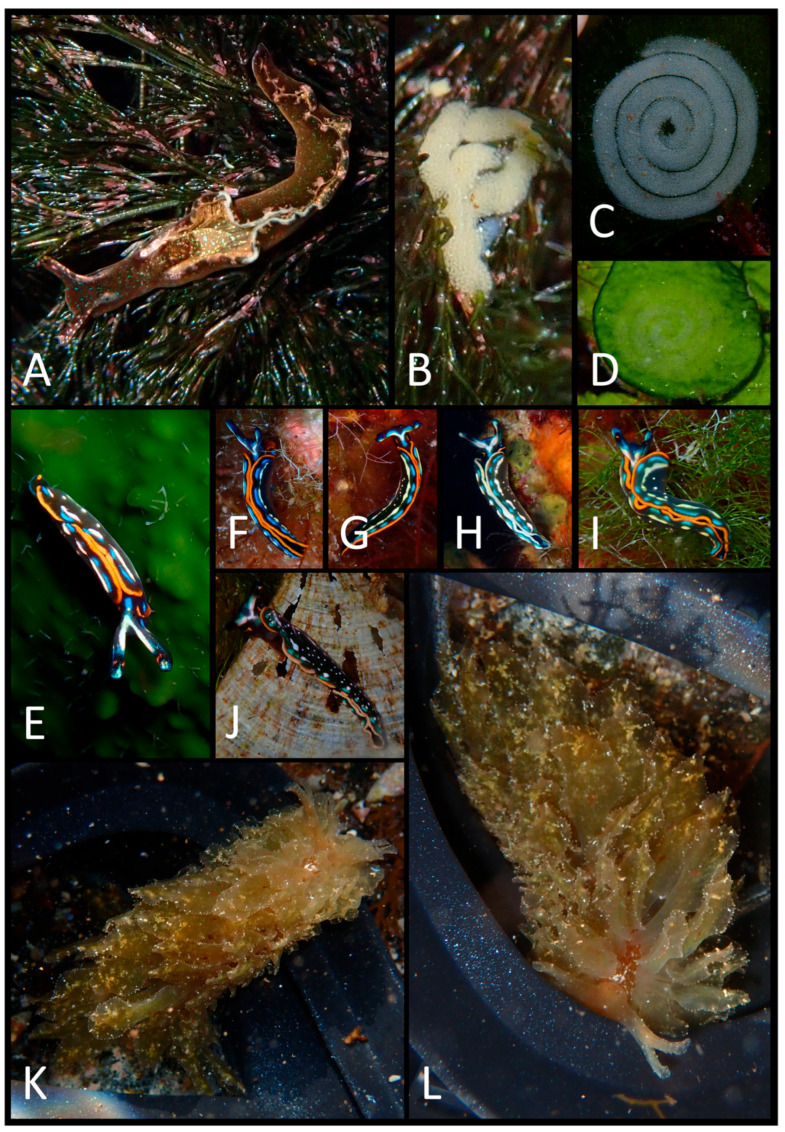
(**A**) *Elysia viridis*; (**B**) *E. viridis*’ egg mass; (**C**) *Elysia* sp.’s egg mass; (**D**) another *Elysia* sp.’s egg mass; (**E**) *Thuridilla hopei*; (**F**) left dorsal-lateral view of *T. hopei*; (**G**) dorsal view *T. hopei*; (**H**) left dorsal–lateral view of *T. hopei*; (**I**) a slightly contracted *T. hopei* specimen; (**J**) a damaged *T. hopei* individual; (**K**) dorsal view of *Caliphylla viridis*; (**L**) anterior–dorsal view of *C. viridis*.

The photographic data collected were used to produce a faunal list (Table 3) illustrated with plates of pictures of live specimens of all species found in the present study (Figure 2, Figure 3, Figure 4, Figure 5, Figure 6, Figure 7 and Figure 8). The taxonomic nomenclature was reported following WoRMS [36]. A comprehensive faunal list with all sea slug species found at Ustica Island to date (literature data and data collected in this study) was produced (Table 1). Finally, a table (Table 4) reporting the types of substrates on which the specimens were found was compiled.

Multivariate analyses were conducted using the software PRIMER 6 with the PERMANOVA+ add-on package. Prior to analysis, species abundance data were square-root-transformed to downweight the contribution of dominant taxa. The Bray–Curtis similarity was used to construct resemblance matrices.

The ordination of samples was performed using principal coordinate analysis (PCoA) to visualise patterns in assemblage structure. Differences among groups were tested using permutational multivariate analysis of variance (PERMANOVA), with 9999 permutations and Type III (partial) sums of squares, using the permutation of residuals under a reduced model. The experimental design included sector, time, and habitat as fixed factors and site as a random factor nested within sector.

The contributions of individual species to within-group similarity and between-group dissimilarity were assessed using similarity percentage (SIMPER) analysis.

## 3. Results

The analysis of the photos allowed us to identify a total of 32 species of sea slugs, belonging to 14 families (Table 3). The taxa presenting the highest numbers of species were Nudibranchia (eight species, four families), Sacoglossa (seven, two), Doridida (six, three), and Aplysiida (five, one), while those with the lowest numbers of species were Rhodopoidea (two, one), Pleurobranchida (two, one), Cephalaspidea (one, one), and Umbraculida (one, one).

Considering the two periods of study, during late spring (T2), a higher number of species was found (25) compared to early autumn (T1) (17). Of these, *Rhodope* sp. 4, *Berthellina* cf. *edwardsii*, *Caloria* cf. *elegans*, *Edmundsella pedata*, *Fiona pinnata*, *Felimida binza*, *Discodorididae* sp., *Tayuva maculosa*, *Haloa* sp., *Aplysia depilans*, *A. fasciata*, *A. punctata*, *Tylodina perversa*, *Elysia viridis*, and *Caliphylla viridis* were documented only in spring. In contrast, *R.* cf. *salviniplaweni*, *Berthella ocellata*, *Doto* sp. 4, *Doto* sp. 5, *Rudmania krohni*, *A. dactylomela*, and *Petalifera petalifera* were found only during autumn. The other species (*Cratena peregrina*, *Flabellina affinis*, *Paraflabellina gabinierei*, *Felimare tricolor*, *Goniodoridella picoensis*, *Bosellia mimetica*, *E. gordanae*, *E. timida*, *Elysia* sp., and *Thuridilla hopei*) were reported in both periods.

**Figure 9:**
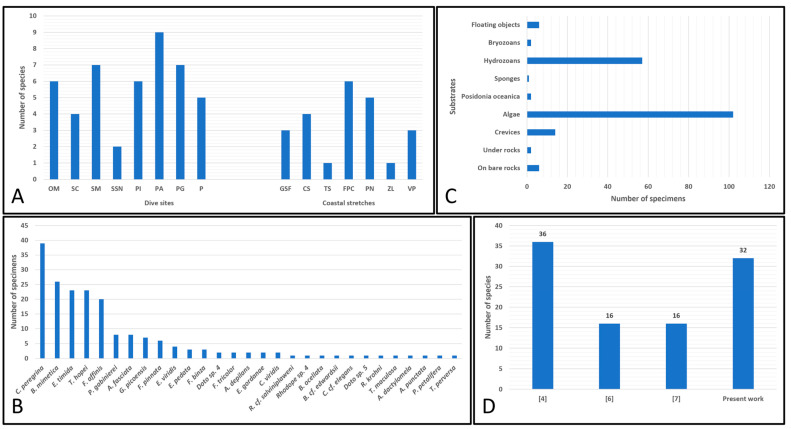
(**A**) Differences in the number of sea slug species between examined dive sites and coastal stretches; (**B**) number of specimens for each found sea slug species documented in this study (*Discodorididae* sp., *Haloa* sp., and *Elysia* sp. were not considered since only the egg masses of these entities were reported); (**C**) numbers of specimens found on the substrate categories considered (*Discodorididae* sp., *Haloa* sp., and *Elysia* sp. were not considered since only the egg masses of these entities were reported); (**D**) differences in the number of sea slug species in historical datasets reported by Chemello [4], Milazzo et al. [6], Milazzo et al. [7] and the present work.

Taking into consideration the eight examined dive sites, the one with the highest number of species was PA (nine species), followed by SM (seven), PG (seven), OM (six), PI (six), P (five) and SC (four). The site with the lowest number of species was SSN (two) (Figure 9A). Regarding the seven coastal stretches, it can be noted that the highest numbers of species were reported at FPC (six species) and PN (five). These were followed by CS (four), GSF (three), VP (three), TS (one), and ZL (one) (Figure 9A). The majority of the documented species presented a very localised distribution, having been found in only one of the island’s sectors (*R.* cf. *salviniplaweni*, *Rhodope* sp. 4, *B. ocellata*, *B.* cf. *edwardsii*, *C.* cf. *elegans*, *F. affinis*, *F. pinnata*, *Doto* sp. 4, *Doto* sp. 5, *F. tricolor*, *R. krohni*, *Discodorididae* sp., *T. maculosa*, *G. picoensis*, *A. dactylomela*, *A. depilans*, *A. punctata*, *P. petalifera*, *T. perversa*, *E. gordanae*, *E. viridis*, and *C. viridis*). Only a few species (*C. peregrina*, *P. gabinierei*, *Haloa* sp., *A. fasciata*, and *Elysia* sp.) were reported in two island sectors. In contrast, *E. pedata*, *F. binza*, *B. mimetica*, *E. timida*, and *T. hopei* were widely distributed across the island, having been found in all sectors (Table 3).

The most abundant species among those found in this study were *C. peregrina* (39 specimens), *B. mimetica* (26), *E. timida* (23), *T. hopei* (23), and *F. affinis* (20). These were followed by *P. gabinerei* (eight), *A. fasciata* (eight), *G. picoensis* (seven), *F. pinnata* (six), *E. viridis* (four), *E. pedata* (three), *F. binza* (three), *Doto* sp. 4 (two), *F. tricolor* (two), *A. depilans* (two), *E. gordanae* (two), and *C. viridis* (two). The remaining species were reported through single findings (*R.* cf. *salviniplaweni*, *Rhodope* sp. 4, *B. ocellata*, *B.* cf. *edwardsii*, *C.* cf. *elegans*, *Doto* sp. 5, *R. krohni*, *T. maculosa*, *A. dactylomela*, *A. punctata*, *P. petalifera*, and *T. perversa*) (Figure 9B).

Regarding the substrates where the documented sea slugs were encountered (Table 4), the majority of specimens were observed on or among algal substrates (102 individuals) (*C.* cf. *elegans*, *C. peregrina*, *E. pedata*, *P. gabinierei*, *F. binza*, *R. krohni*, *T. maculosa*, *A. depilans*, *A. fasciata*, *A. punctata*, *T. perversa*, *B. mimetica*, *E. gordanae*, *E. timida*, *E. viridis*, *T. hopei*, and *C. viridis*). Most of the animals not found on or among this type of substrate were recorded on hydrozoans (57) (*C. peregrina*, *F. affinis*, *P. gabinierei*, *Doto* sp. 4, and *Doto* sp. 5), within crevices (14) (*B. ocellata*, *E. pedata*, *P. gabinierei*, *F. tricolor*, *F. binza*, *G. picoensis*, and *T. hopei*), on bare rocky substrates (six) (*A. dactylomela*, *E. timida*, and *T. hopei*), and on floating objects (six) (*F. pinnata*). A minority of individuals were found under rocks (two) (*R. salviniplaweni* and *B.* cf. *edwardsii*), on *Posidonia oceanica* leaves or rhizomes (two) (*E. pedata* and *P. petalifera*), on bryozoans (two) (*G. picoensis*), and on sponges (one) (*Rhodope* sp. 4) (Figure 9C).

To quantitatively assess patterns of similarity in taxonomic composition across spatial and temporal scales, multivariate analyses based on the Bray–Curtis similarity were performed.

**Figure 10:**
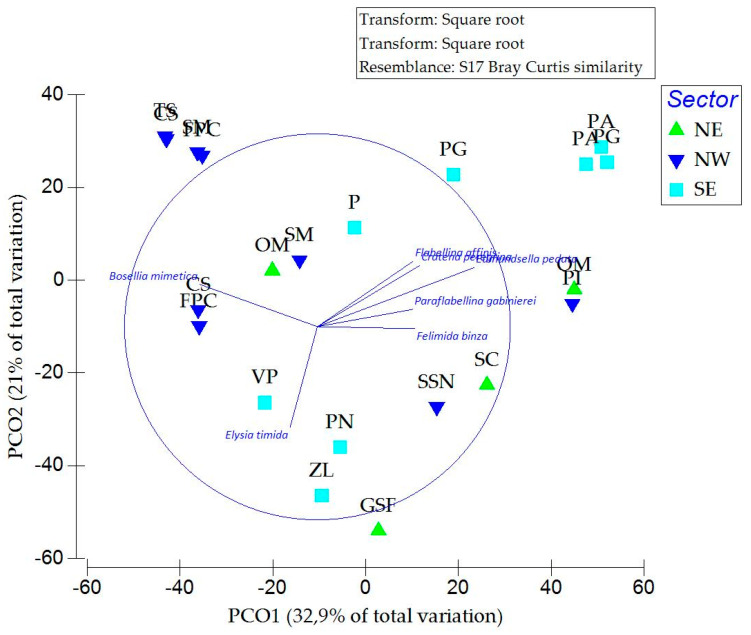
Principal coordinate analysis (PCoA) based on abundance data divided by sector. The most significant species are included to highlight differences among sectors.

Principal coordinate analysis (PCoA), based on square-root-transformed abundance data, explained 53.9% of the total variation along the first two axes (PCO1: 32.9%; PCO2: 21%) (Figure 10). The ordination revealed the clear spatial structuring of sea slug assemblages, with samples primarily distributed along PCO1 according to the island sector. Although partial overlap among sectors was observed, distinct groupings indicated substantial differences in assemblage composition at the spatial scale considered.

Temporal and habitat-related patterns were less evident in the ordination, as samples from different time points and habitats largely overlapped within sectors, suggesting that these factors contributed less to the overall variability than spatial differences.

The PERMANOVA confirmed these patterns, revealing significant effects of sector (pseudo-F = 2.24, *p* = 0.048), time (pseudo-F = 3.53, *p* = 0.011), and habitat (pseudo-F = 3.07, *p* = 0.016) on the assemblage structure. Spatial differences among sectors were statistically supported, confirming that the observed variation in species richness among sites reflected underlying differences in community composition.

In contrast to the descriptive patterns previously observed, the PERMANOVA detected a significant effect of time, indicating that seasonal differences in assemblage composition were statistically supported, despite being less evident in the ordination space. No significant interactions among factors were detected (*p* > 0.05), indicating that the effects of time and habitat were consistent across sectors.

The SIMPER analysis further supported these patterns by identifying the species contributing most to similarities within groups and dissimilarities among sectors. Within-sector similarity was relatively low (NE: 31.6%, NW: 25.0%, SE: 22.1%), indicating considerable variability in assemblage composition. The NE sector was characterised by *T. hopei*, which accounted for the majority of within-group similarity, while the NW sector was dominated by *B. mimetica*. In contrast, the SE sector displayed a more heterogeneous assemblage, with several species contributing to within-group similarity, including *C. peregrina*, *F. affinis*, *G. picoensis*, and *E. timida*.

Pairwise comparisons indicated high dissimilarity among sectors (NE vs. NW: 81.2%; NE vs. SE: 80.3%; NW vs. SE: 86.5%), driven primarily by differences in the relative abundance of key taxa such as *T. hopei*, *B. mimetica*, *C. peregrina*, and *F. affinis*.

## 4. Discussion

The results of the present study seem to confirm the model of species distribution hypothesised by Riggio and Milazzo [3] for the coasts of Ustica Island. According to these authors, the island sectors affected by the south/west sea currents would be those that present the highest marine diversity. This is precisely the situation detected in this study, with the north-west and south-east sectors presenting the highest numbers of sea slug species. Indeed, the dive sites (Punta dell’Arpa, Punta Galera, and Scoglio del Medico) and the coastal stretches (Faro di Punta Cavazzi, Piscina Naturale, and Cala Sidoti) located exactly in these sectors showed the highest numbers of species.

Nevertheless, this situation can only be explained at the level of individual sectors (macroscale). In fact, if we look in detail at the individual dive sites and coastal stretches (microscale), the species richness exhibited some degree of spatial heterogeneity. Consequently, although south-west sea currents would appear to be the main source of allochthonous larvae for this island (most sea slugs have planktotrophic veliger larvae [29,37]), they alone cannot explain the differences in species number found between the various dive sites and coastal stretches examined.

In this regard, the environmental conditions of individual locations would play a very important role in determining the numerical difference in sea slug species. The great importance of environmental conditions suitable for sea slugs has already been highlighted for the seabeds and coastlines of other Sicilian islands. Specifically, it has been noted that most sea slugs prefer habitats characterised by coralligenous biocenoses rich in benthic suspension feeders (e.g., sponges, bryozoans, cnidarians, and ascidians), which are the main prey of these molluscs. At the same time, in very shallow environments, sea slugs tend to favour shelter areas with calm waters [26,27]. This rule of thumb seems to apply well to the locations examined in this study. In fact, considering the dive sites, those with the highest numbers of species are precisely those that feature the above-mentioned environments. Specifically, Punta dell’Arpa (the site with the highest number of species) has a striking coralligenous biocoenosis that is more or less rich in suspension benthic feeders. Scoglio del Medico (the site with the second-highest number of species), while not having such a developed coralligenous biocoenosis, is characterised by a large number of crevices that host many sciaphilous algae and benthic suspension feeders. The other examined dive sites (presenting a lower number of species) did not present such a variety of environmental types. As regards the coastal stretches examined, only those of Faro di Punta Cavazzi and Piscina Naturale (the richest in species) are located within sheltered areas with relatively calm waters. All the other coastal stretches (almost all of which had a lower number of species) are located on highly exposed coasts. The Cala Sidoti site (with an intermediate number of species) presents an intermediate situation between a sheltered and an exposed area.

Through the comparison of the data reported here with those from the literature on the sea slugs of Ustica Island (Figure 9D), it is interesting to note that the current data are very similar in value to those reported by Chemello [4] almost 40 years ago. The slightly higher values reported by Chemello [4] were probably caused by the data collection method (destructive with substrate collection), which most likely allowed the reporting of a slightly higher number of species than that found with the technique implemented in this study (non-destructive and exclusively photographic). Therefore, this might suggest that the photographic capture technique could provide similar quantitative results to those provided by the destructive method. Meanwhile, the differences between the species richness reported here and that published by Milazzo et al. [6,7] are probably because, in the latter studies, a smaller number of sites was surveyed. Consequently, the sampling effort was significantly lower.

**Figure 11:**
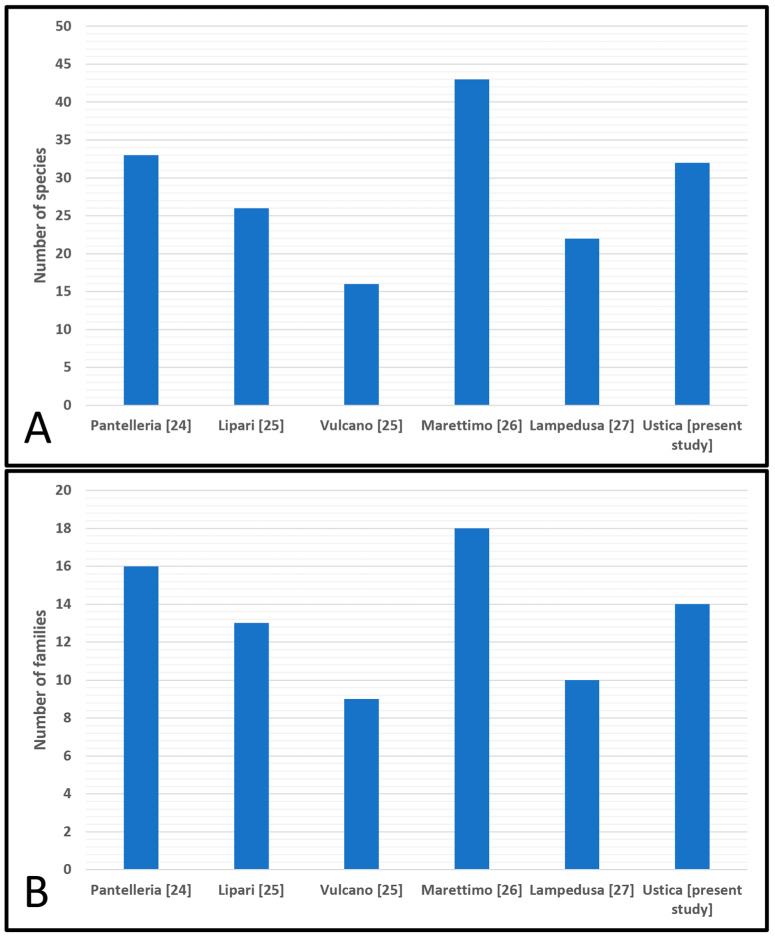
(**A**) Differences in the number of species between the present study and the other Sicilian islands recently surveyed with the same methodology: Pantelleria–Lombardo and Marletta [24]; Lipari and Vulcano–Lombardo and Marletta [25]; Marettimo–Lombardo and Marletta [26]; Lampedusa–Lombardo and Marletta [27]; (**B**) differences in the number of families between the present study and the other Sicilian islands recently surveyed with the same methodology: Pantelleria–Lombardo and Marletta [24]; Lipari and Vulcano–Lombardo and Marletta [25]; Marettimo–Lombardo and Marletta [26]; Lampedusa–Lombardo and Marletta [27].

Comparing the sea slug fauna found in Ustica Island through this study with those found in other Sicilian islands recently studied using the same method [24,25,26,27], it can be seen that the number of species found on this island is different from that expected. In fact, considering Ustica’s presumed role as a hotspot of marine biodiversity within the Mediterranean basin [3], this island should have a richer sea slug fauna than the other explored Sicilian islands. However, by comparing the number of species found in this study with those observed in other Sicilian islands recently studied (Figure 10), it can be observed that the sea slug fauna of Ustica is not as rich as previously supposed. Indeed, in this study, a total of 32 species and 14 families were identified through two different campaigns, while, in the other investigated Sicilian islands [24,25,26,27], only through a single sampling campaign, a similar or even higher number of species and families was found (Figure 11A,B).

Of the 32 species found during this study, nine of them (*Rhodope* cf. *salviniplaweni*, *Rhodope* sp. 4, *Berthellina* cf. *edwardsii*, *Paraflabellina gabinierei*, *Fiona pinnata*, *Felimida binza*, *Petalifera petalifera*, *Elysia gordanae*, and *Caliphylla viridis*) appear to be new reports for the island. Even the five undetermined taxa (*Doto* sp. 4, *Doto* sp. 5, *Discodorididae* sp., *Haloa* sp., and *Elysia* sp.) seem to be new findings for Ustica Island. Nevertheless, considering that we do not know the external morphologies of *Dotoidae* sp. 1 and *Dotoidae* sp. 2, reported in the first list of Ustica’s gastropods created by Chemello [4], and that the present findings of *Discodorididae* sp., *Haloa* sp., and *Elysia* sp. were discovered through egg masses, we cannot be sure that these entities have not already been found on the island (see Table 1).

Considering the new findings, particularly interesting are those of the rhodopoid *R.* cf. *salviniplaweni*, the nudibranchs *P. gabinierei* and *Fiona pinnata*, and the sacoglossan *C. viridis*.

The species here reported as *R.* cf. *salviniplaweni* presented remarkable similarity to the taxon described in Fernández-Simón et al. [33]. In fact, the specimen that we found had an orange dorsal patch covering most of the central body surface (two thirds of the notum). This finding, almost certainly attributable to this taxonomic entity, represents the second Mediterranean record of this species. In fact, since its description, *R. salviniplaweni* has been found exclusively in certain locations on the north-east coast of Spain (Cadaqués) [33].

*Paraflabellina gabinierei* is a species that is endemic to the Mediterranean Sea for which very little is known about its biology. At present, the sporadic findings of this species are derived from reports from Turkey, Israel, Croatia, France, Spain, and Italy [38]. In Sicily, *P. gabinierei* was found only on the islands of Lipari, Vulcano [25] and Marettimo [26]. At Ustica Island, this nudibranch was documented at Piramidi, Punta dell’Arpa, and Punta Galera. At Piramidi, five individuals were found during late spring: a single specimen on filamentous red algae (12.4 m, 22 °C) and a group of four animals on a shaded vertical wall on a turf of *Halopteris filicina* (Grateloup) Kützing and *Jania* sp. with sparse thalli of *Dictyota* sp., *Padina* sp., *Zonaria tournefortii* (J.V.Lamouroux) Montagne, *Flabellia petiolata* (Turra) Nizamuddin, and bryozoans (13.7 m, 22 °C). In the dive site of Punta dell’Arpa, two individuals were found: one during early autumn on a turf of filamentous red algae intermingled with *Jania* sp., *Dictyota* sp., and erect bryozoans (21.9 m, 24 °C) and the other in late spring on a thallus of the brown algae *Ericaria funkii* (Gerloff & Nizamuddin) Molinari & Guiry (35 m, 16 °C), strongly covered by hydrozoans. At Punta Galera, only one specimen was documented during late spring on a shaded vertical wall covered with *Peyssonnelia* spp. and filamentous red algae (12.2 m, 23 °C).

The present report of *F. pinnata* for Ustica Island is remarkable since, until now, the findings of this species in Sicilian waters referred only to the area of the province of Catania [10]. This scarcity of findings in Sicily, considering that *F. pinnata* is a cosmopolitan species that lives on floating objects colonised by the barnacle *Lepas* spp. [11], once again highlights the historical lack of experts and specific studies dedicated to these animals in Sicily [10]. At Ustica, this species was documented through the finding of six *F. pinnata* individuals (with many egg masses) on a floating sack covered with a large number of *Lepas* spp.

Considering the last species, *C. viridis*, the finding of a pair of individuals at Faro di Punta Cavazzi (late spring) among several thalli of *Sargassum vulgare* C. Agardh in a few cm of water represents the third photographic report of this sacoglossan for the Mediterranean basin. Indeed, to date, this rare species has been found only on some Mediterranean islands. Specifically, this sacoglossan was found at Rhodos (Greece), Gozo (Malta) [39], Pantelleria (as *Polybranchia* sp.) [24], and Lampedusa (as *Polybranchia* sp.) [27]. It is interesting to note that all these reports were documented in late spring (June 2017 [39] and present report) or the summer months (July 2022 and 2025 [24,27]).

The use of multivariate analyses based on the Bray–Curtis similarity provided a robust statistical framework supporting the previously observed descriptive patterns of spatial and seasonal variability. These results strengthen the interpretation of assemblage differences, demonstrating that both spatial and temporal variations are not only apparent in species richness but also reflected in the overall assemblage structure.

The present study highlights the importance of spatial variability in structuring sea-slug assemblages, with the coastal sector emerging as the primary driver of community differentiation. The separation observed in the ordination, together with the significant PERMANOVA results, suggests that broad-scale spatial processes, such as environmental gradients or habitat distribution, play a key role in shaping species composition.

Although temporal variation was not strongly evident in the ordination, the PERMANOVA detected a significant effect of time, indicating that temporal changes in assemblage structure do occur but are comparatively subtle. This discrepancy likely reflects the different sensitivities of ordination and hypothesis-testing approaches, with the former emphasizing dominant gradients and the latter detecting consistent but less pronounced effects.

Habitat also contributed significantly to the assemblage structure, suggesting that local environmental conditions influence species distribution. However, the absence of significant interactions indicates that habitat effects are relatively consistent across sectors and time, rather than context-dependent.

The SIMPER analysis further revealed that differences among sectors were largely driven by a limited number of key species. The dominance of *Thuridilla hopei* in the NE sector and *Bosellia mimetica* in the NW sector indicated the strong spatial structuring of characteristic taxa, potentially linked to differences in resource availability or habitat features. In contrast, the SE sector exhibited a more diverse assemblage, with multiple species contributing to the community composition, suggesting higher environmental heterogeneity or niche availability.

Overall, these findings indicate that molluscan assemblages are structured hierarchically, with spatial variability acting as the primary driver, while temporal and habitat-related factors contribute to secondary, finer-scale variation.

## 5. Conclusions

Through the current faunal data presented here and the examination of pre-existing data, it can be seen that, since the publication of the first malacological work dedicated to Ustica Island [4], to date, a total of 77 species and 33 families of sea slugs have been found on the island’s seabed (Table 1). This level of biodiversity is currently the highest found on a Sicilian island for the informal group of sea slugs [24,25,26,27]. However, this high value is mostly due to the fact that Ustica is the Sicilian island for which the longest historical series of malacological data exists. In this regard, considering the faunal richness documented for the island [3], it would be interesting in the future to carry out similar studies on other groups of marine invertebrates to evaluate whether this richness is only due to the collection of historical data or to frequent monitoring activities, which could provide updated information on the biodiversity of Ustica Island.

## Figures and Tables

**Table 1:** List of all sea slug species found at Ustica Island to date (literature data and data collected in the present study).

Taxon	Literature Data	Present Study	No. of Species	No. of Families
**Superfamily Rhodopoidea Ihering, 1876**			2	1
**Family Rhodopidae Ihering, 1876**				
*Rhodope* cf. *salviniplaweni* Fernández-Simón & Moles, 2025		x		
*Rhodope* sp. 4		x		
**Order Pleurobranchida Gray, 1827**			3	1
**Family Pleurobranchidae Gray, 1827**				
*Berthella aurantiaca* (Risso, 1818)	[4,14]			
*Berthella ocellata* (Delle Chiaje, 1830)	[4,14]	x		
*Berthellina* cf. *edwardsii* (Vayssière, 1897)		x		
**Order Nudibranchia Blainville, 1814**			19	10
**Family Aeolidiidae J. E. Gray, 1827**				
*Aeolidiella alderi* (Cocks, 1852)	[4]			
**Family Facelinidae Bergh, 1889**				
*Caloria elegans* (Alder & Hancock, 1845)	[14]			
*Caloria* cf. *elegans* (Alder & Hancock, 1845)		x		
*Cratena peregrina* (Gmelin, 1791)	[4,14]	x		
**Family Flabellinidae Bergh, 1889**				
*Edmundsella pedata* (Montagu, 1816)	[4,14,15]	x		
*Flabellina affinis* (Gmelin, 1791)	[4,14,15]	x		
*Paraflabellina gabinierei* (Vicente, 1975)		x		
**Family Coryphellidae Bergh, 1889**				
*Fjordia lineata* (Lovén, 1846)	[4,14]			
**Family Fionidae J. E. Gray, 1857**				
*Fiona pinnata* (Eschscholtz, 1831)		x		
**Family Trinchesiidae F. Nordsieck, 1972**				
*Trinchesia caerulea* (Montagu, 1804)	[4,6,7,14]			
**Family Dotidae Gray, 1853**				
*Doto* cf. *fragilis* (Forbes, 1838)	[4]			
*Doto* sp. 4		x		
*Doto* sp. 5		x		
*Dotoidae* sp. 1	[14]			
*Dotoidae* sp. 2	[14]			
**Family Tritoniidae Lamarck, 1809**				
*Candiella lineata* (Alder & Hancock, 1848)	[4,6,7]			
*Candiella striata* (Haefelfinger, 1963)	[14]			
**Family Janolidae Pruvot-Fol, 1933**				
*Antiopella cristata* (Delle Chiaje, 1841)	[4,14]			
**Family Embletoniidae Pruvot-Fol, 1954**				
*Embletonia pulchra* (Alder & Hancock, 1844)	[8]			
**Order Doridida Pelseneer, 1894**			22	8
**Family Chromodorididae Bergh, 1891**				
*Felimare picta* (R. A. Philippi, 1836)	[4,14,15]			
*Felimare tricolor* (Cantraine, 1835)	[4,14]	x		
*Felimare villafranca* (Risso, 1818)	[4,14]			
*Felimare* cf. *villafranca* (Risso, 1818)	[4]			
*Felimida binza* (Ev. Marcus & Er. Marcus, 1963)		x		
*Rudmania krohni* (Verany, 1846)	[4,14]	x		
*Rudmania purpurea* (Risso, 1831)	[14]			
*Rudmania* cf. *purpurea* (Risso, 1831)	[4]			
**Family Discodorididae Bergh, 1891**				
*Peltodoris atromaculata* Bergh, 1880	[4,14]			
*Platydoris argo* (Linnaeus, 1767)	[4,14]			
*Tayuva maculosa* (Bergh, 1884)	[6,7]	x		
*Discodorididae* sp.		x		
**Family Dorididae Rafinesque, 1815**				
*Doris verrucosa* Linnaeus, 1758	[4,14]			
**Family Aegiridae P. Fischer, 1883**				
*Aegires punctilucens* (A. d’Orbigny, 1837)	[8]			
**Family Goniodorididae H. Adams & A. Adams, 1854**				
*Goniodoridella picoensis* (Paz-Sedano, Ortigosa & Pola, 2017)	[16]	x		
*Pelagella castanea* (Alder & Hancock, 1845)	[6,7]			
*Trapania maculata* Haefelfinger, 1960	[15]			
**Family Dendrodorididae O’Donoghue, 1924 (1864)**				
*Dendrodoris grandiflora* (Rapp, 1827)	[4,14]			
*Doriopsilla areolata* Bergh, 1880	[6,7]			
**Family Phyllidiidae Rafinesque, 1814**				
*Phyllidia flava* Aradas, 1847	[4,14]			
**Family Polyceridae Alder & Hancock, 1845**				
*Palio dubia* (M. Sars, 1829)	[6,7]			
*Polycera quadrilineata* (O. F. Müller, 1776)	[14,15]			
**Order Cephalaspidea P. Fischer, 1883**			13	7
**Family Bullidae J. E. Gray, 1827**				
*Bulla striata* Bruguière, 1792	[4]			
**Family Retusidae Thiele, 1925**				
*Retusa mammillata* (R. A. Philippi, 1836)	[4]			
*Retusa truncatula* (Bruguière, 1792)	[4,6,7,8]			
*Retusa* cf. *truncatula* (Bruguière, 1792)	[4]			
**Family Cylichnidae H. Adams & A. Adams, 1854**				
*Cylichna cylindracea* (Pennant, 1777)	[4]			
**Family Diaphanidae Odhner, 1914 (1857)**				
*Diaphana minuta* T. Brown, 1827	[7]			
**Family Haminoeidae Pilsbry, 1895**				
*Haloa* sp.		x		
*Haminoea hydatis* (Linnaeus, 1758)	[6,7]			
*Lamprohaminoea ovalis* (Pease, 1868)	[18]			
*Roxaniella jeffreysi* (Weinkauff, 1866)	[6,7]			
**Family Aglajidae Pilsbry, 1895 (1847)**				
*Camachoaglaja africana* (Pruvot-Fol, 1953)	[17]			
**Family Philinidae J. E. Gray, 1850 (1815)**				
*Philine aperta* (Linnaeus, 1767)	[4]			
*Philine catena* (Montagu, 1803)	[4,6,7]			
**Order Runcinida Colosi, 1918**			3	1
**Family Runcinidae H. Adams & A. Adams, 1854**				
*Runcina* sp. 1	[6]			
*Runcina* sp. 2	[6]			
*Runcina* sp.	[7]			
**Order Aplysiida P. Fischer, 1883**			6	1
**Family Aplysiidae Lamarck, 1809**				
*Aplysia dactylomela* Rang, 1828	[17]	x		
*Aplysia depilans* Gmelin, 1791	[4,14]	x		
*Aplysia fasciata* Poiret, 1789	[4,6,7,14]	x		
*Aplysia punctata* (Cuvier, 1803)	[4,6,7,8,14]	x		
*Petalifera petalifera* (Rang, 1828)		x		
*Phyllaplysia lafonti* (P. Fischer, 1870)	[6,7]			
**Order Umbraculida Odhner, 1939**			2	2
**Family Tylodinidae Gray, 1847**				
*Tylodina perversa* (Gmelin, 1791)	[4,14]	x		
**Family Umbraculidae Dall, 1889 (1827)**				
*Umbraculum umbraculum* ([Lightfoot], 1786)	[14]			
**Superorder Sacoglossa Ihering, 1876**			7	2
**Family Plakobranchidae J. E. Gray, 1840**				
*Bosellia mimetica* Trinchese, 1891	[4,14]	x		
*Elysia gordanae* T. E. Thompson & Jaklin, 1988		x		
*Elysia timida* (Risso, 1818)	[4,14]	x		
*Elysia viridis* (Montagu, 1804)	[6,7]	x		
*Elysia* sp.		x		
*Thuridilla hopei* (Vérany, 1853)	[4,5,14]	x		
**Family Caliphyllidae Tiberi, 1881**				
*Caliphylla viridis* (Deshayes, 1857)		x		
**Total number of species and families**			77	33

**Table 2:** List of the examined dive sites and coastal stretches. For each one, information about the locality name, used abbreviation, locality description, coordinates, and date/s of activity is given. Regarding the latter, the dates on the left and on the right of “;” refer to the early autumn (T1) and late spring (T2) surveys, respectively. The letters “nd” (i.e., not done) indicate that, for that season, it was not possible to examine the locality in question.

Locality	Abbreviation	Description	Coordinates	Date/s
**Dive Sites**				
Omo Morto	OM	A slope of boulders mixed with *Posidonia oceanica* (Linnaeus) Delile, 1813 meadows, flanking an enormous vertical rocky wall. The latter rests on a flat sandy seabed (at a depth of about 30 m). The rocky wall has a cave at about 25 m.	38°42′41.1″ N 13°12′00.1″ E	27 September 2024; 12 June 2025
Secca della Colombara	SC	A gigantic quadrangular rocky outcrop that starts at 2–3 m and extends up to 45–50 m. This structure is flanked by smaller rock formations and the remains of a shipwreck, which together form numerous crevices of various sizes.	38°43′48.7″ N 13°10′48.6″ E	nd; 11 June 2025
Scoglio del Medico	SM	A gigantic rocky outcrop whose peak rises out of the water. The submerged part is characterised by countless walls, caves, ravines, and crevices. The outcrop rises from a flat sandy seabed at a depth of about 50 m.	38°42′55.6″ N 13°09′21.0″ E	1 October 2024; 10 June 2025
Sito Senza Nome	SSN	Very uniform and shallow seabed (8–10 m) characterised by rocks, boulders, and pebbles.	38°42′22.6″ N 13°09′18.4″ E	1 October 2024; nd
Piramidi	PI	A group of three rocky outcrops resting on a seabed at a depth of approximately 22–25 m. These outcrops (which start at a depth of 3 m) feature small canyons and walls between them.	38°41′56.5″ N 13°09′05.3″ E	nd; 13 June 2025
Punta dell’Arpa	PA	A wide *P. oceanica* meadow that ends at about 13 m, giving space to a rocky escarpment that falls to a depth of about 40 m. The latter has rocky outcrops characterised by sciaphilous coralligenous communities and *Paramuricea clavata* (Risso, 1827).	38°41′26.1″ N 13°10′26.0″ E	30 September 2024; 12 June 2025
Punta Galera	PG	Large rocky wall parallel to the coastline, resting on a sandy seabed at a depth of 30–35 m. The shallower part of the site is characterised by a pebble bed and a meadow of *P. oceanica*.	38°41′40.4″ N 13°10′59.6″ E	30 September 2024; 14 June 2025
Pastizza	P	Large rocky wall (rich in crevices) parallel to the coast, resting on a sandy seabed characterised by *P. oceanica* at a depth of about 25 m. The shallowest area of the site features a rocky escarpment and scattered boulders.	38°42′07.5″ N 13°11′35.6″ E	nd; 10 June 2025
**Coastal stretches**				
Gorgo Salato-Faraglione	GSF	A gulf characterised by shallow waters (1 m) with boulders and pebbles sloping down towards the sea. The coastline features jagged cliffs and a large sea stack.	From 38°43′13.8″ N 13°10′51.7″ E to 38°43′09.9″ N 13°10′58.7″ E	1 October 2024; nd
Cala Sidoti	CS	Very shallow area (0.5–1 m) rich in rocks and pebbles, characterised by rocky outcrops that form many inlets and narrow connecting channels.	From 38°42′26.9″ N 13°09′36.7″ E to 38°42′23.5″ N 13°09′33.7″ E	28 September 2024; 13 June 2025
Torre Spalmatore	TS	A cove characterised by cliffs that drop to a depth of about 15 m, with boulders and rocks on the seabed.	From 38°41′59.5″ N 13°09′12.0″ E to 38°42′02.1″ N 13°09′09.4″ E	28 September 2024; nd
Faro di Punta Cavazzi	FPC	A small bay characterised by a shallow reef (0–1 m) with boulders and small pebbles. The innermost part features a conspicuous concrete platform that has been deposited on top of the rocky plateau. The area also has a small natural pool almost completely separated from the sea, characterised by volcanic ash, stones, pebbles, and organic debris.	From 38°41′41.2″ N 13°09′16.5″ E to 38°41′40.1″ N 13°09′16.0″ E	28 September 2024; 11 June 2025
Piscina Naturale	PN	An inlet forming a natural pool (approximately 8 m deep) that connects to the outside mainly through a short underwater tunnel. The funnel-shaped pool has numerous small inlets and puddles almost at water level.	From 38°41′37.6″ N 13°09′16.2″ E to 38°41′37.2″ N 13°09′15.2″ E	nd; 14 June 2025
Zia Lisa	ZL	Cove with a steep seabed and rocky outcrops that slope down to a depth of 6–10 m.	From 38°41′30.5″ N 13°09′46.5″ E to 38°41′31.4″ N 13°09′43.4″ E	nd; 11 June 2025
Villaggio dei Pescatori	VP	A jagged coastline characterised by an almost total absence of vegetation. The cliffs drop almost immediately to a depth of more than 16 m. This area features a large submerged cave.	From 38°42′31.2″ N 13°11′53.2″ E to 38°42′34.9″ N 13°12′02.7″ E	29 September 2024; nd

**Table 3:** List of the sea slug species found during this study. For each species, the locality of finding and the number of found specimens are indicated. The numbers on the left and on the right with respect to “;” indicate the early autumn (T1) and late spring surveys (T2), respectively. The letters “nd” (i.e., not done) indicate that, for that season, it was not possible to examine the locality in question. The symbols “*” and “‡” indicate the observation of egg masses and mating activities, respectively. The location abbreviations are indicated in Table 2.

	North-East Sector			North-West Sector						South-East Sector						
	C Zone	B Zone					A Zone			C Zone						
Taxon	OM	SC	GSF	FPC	PI	SM	TS	SSN	CS	PN	ZL	PA	PG	P	VP	Depth Range
**Superfamily Rhodopoidea Ihering, 1876**																
**Family Rhodopidae Ihering, 1876**																
*Rhodope* cf. *salviniplaweni* Fernández-Simón & Moles, 2025				1; 0												1 < m
*Rhodope* sp. 4		nd; 1														14.1 m
**Order Pleurobranchida Gray, 1827**																
**Family Pleurobranchidae Gray, 1827**																
*Berthella ocellata* (Delle Chiaje, 1830)												1; 0				26.7 m
*Berthellina* cf. *edwardsii* (Vayssière, 1897)									0; 1							1 < m
**Order Nudibranchia Blainville, 1814**																
**Family Facelinidae Bergh, 1889**																
*Caloria* cf. *elegans* (Alder & Hancock, 1845)						0; 1										24 m
*Cratena peregrina* (Gmelin, 1791)	0; 5 *											3; 3	6 *; 22 *			10–25.1 m
**Family Flabellinidae Bergh, 1889**																
*Edmundsella pedata* (Montagu, 1816)	0; 1				nd; 1							0; 1				10.8–30.6 m
*Flabellina affinis* (Gmelin, 1791)												4; 1	3 *; 12 *‡			9.3–29.5 m
*Paraflabellina gabinierei* (Vicente, 1975)					nd; 5							1; 1	0; 1			12.2–35 m
**Family Fionidae J. E. Gray, 1857**																
*Fiona pinnata* (Eschscholtz, 1831)														nd; 6 *		1 < m
**Family Dotidae Gray, 1853**																
*Doto* sp. 4						2; 0										6.3 m
*Doto* sp. 5												1 *; 0				1.3 m
**Order Doridida Pelseneer, 1894**																
**Family Chromodorididae Bergh, 1891**																
*Felimare tricolor* (Cantraine, 1835)												1; 0		nd; 1		24.7–35.1 m
*Felimida binza* (Ev. Marcus & Er. Marcus, 1963)		nd; 1			nd; 1								0; 1			11.8–22 m
*Rudmania krohni* (Verany, 1846)	0; 1															11.3 m
**Family Discodorididae Bergh, 1891**																
*Discodorididae* sp.										nd; 0 *						1 < m
*Tayuva maculosa* (Bergh, 1884)									0; 1							1 < m
**Family Goniodorididae H. Adams & A. Adams, 1854**																
*Goniodoridella picoensis* (Paz-Sedano, Ortigosa & Pola, 2017)												0; 1	1; 2	nd; 3		8.4–38.7 m
**Order Cephalaspidea P. Fischer, 1883**																
**Family Haminoeidae Pilsbry, 1895**																
*Haloa* sp.	0; 0 *	nd; 0 *			nd; 0 *	0; 0 *										9.8–24.4 m
**Order Aplysiida P. Fischer, 1883**																
**Family Aplysiidae Lamarck, 1809**																
*Aplysia dactylomela* Rang, 1828			1; nd													1 < m
*Aplysia depilans* Gmelin, 1791										nd; 2 *‡						1 < m
*Aplysia fasciata* Poiret, 1789						0; 3				nd; 5 *‡						16–20.8 m
*Aplysia punctata* (Cuvier, 1803)				0; 1												
*Petalifera petalifera* (Rang, 1828)				1; 0												1 < m
**Order Umbraculida Odhner, 1939**																
**Family Tylodinidae Gray, 1847**																
*Tylodina perversa* (Gmelin, 1791)						0; 1										16 m
**Superorder Sacoglossa Ihering, 1876**																
**Family Plakobranchidae Gray, 1840**																
*Bosellia mimetica* Trinchese, 1891	1; 0			2; 2		1 *; 2	2 *; nd		4; 8				1; 0	nd; 2	1; nd	1 < −24.7 m
*Elysia gordanae* T. E. Thompson & Jaklin, 1988						0; 1		1; nd								5.8–19.5 m
*Elysia timida* (Risso, 1818)			3; nd	0; 2					0; 1	nd; 12	nd; 1				4; nd	1 < m
*Elysia viridis* (Montagu, 1804)										nd; 4 *						1 < m
*Elysia* sp.					nd; 0 *							0 *; 0				15.5–32.3 m
*Thuridilla hopei* (Vérany, 1853)	2; 2	nd; 1	1; nd		nd; 3			2; nd					2; 0	nd; 3	7; nd	1 < −27.2 m
**Family Caliphyllidae Tiberi, 1881**																
*Caliphylla viridis* (Deshayes, 1857)				0; 2												1 < m
**Total number of species per site**	6	4	3	6	6	7	1	2	4	5	1	9	7	5	3	
**Total number of species**	32															
**Total number of families per site**	5	4	2	4	4	6	1	1	3	3	1	7	5	4	1	
**Total number of families**	14															

**Table 4:** The list shows the substrates on which the specimens were found, as well as the number of specimens recorded for that substrate for each species observed. *Discodorididae* sp., *Haloa* sp., and *Elysia* sp. were not included, as only the egg masses of these organisms were reported.

Taxon	Substrate								
	On Bare Rocks	Under Rocks	Crevices	Algae	Posidonia oceanica	Sponges	Hydrozoans	Bryozoans	Floating Objects
*Rhodope* cf. *salviniplaweni* Fernández-Simón & Moles, 2025		1							
*Rhodope* sp. 4						1			
*Berthella ocellata* (Delle Chiaje, 1830)			1						
*Berthellina* cf. *edwardsii* (Vayssière, 1897)		1							
*Caloria* cf. *elegans* (Alder & Hancock, 1845)				1					
*Cratena peregrina* (Gmelin, 1791)				6			33		
*Edmundsella pedata* (Montagu, 1816)			1	1	1				
*Flabellina affinis* (Gmelin, 1791)							20		
*Paraflabellina gabinierei* (Vicente, 1975)			1	6			1		
*Fiona pinnata* (Eschscholtz, 1831)									6
*Doto* sp. 4							2		
*Doto* sp. 5							1		
*Felimare tricolor* (Cantraine, 1835)			2						
*Felimida binza* (Ev. Marcus & Er. Marcus, 1963)			2	1					
*Rudmania krohni* (Verany, 1846)				1					
*Tayuva maculosa* (Bergh, 1884)				1					
*Goniodoridella picoensis* (Paz-Sedano, Ortigosa & Pola, 2017)			2	3				2	
*Aplysia dactylomela* Rang, 1828	1								
*Aplysia depilans* Gmelin, 1791				2					
*Aplysia fasciata* Poiret, 1789				8					
*Aplysia punctata* (Cuvier, 1803)				1					
*Petalifera petalifera* (Rang, 1828)					1				
*Tylodina perversa* (Gmelin, 1791)				1					
*Bosellia mimetica* Trinchese, 1891				26					
*Elysia gordanae* T. E. Thompson & Jaklin, 1988				2					
*Elysia timida* (Risso, 1818)	1			22					
*Elysia viridis* (Montagu, 1804)				4					
*Thuridilla hopei* (Vérany, 1853)	4		5	14					
*Caliphylla viridis* (Deshayes, 1857)				2					
**Total number of specimens per substrate type**	6	2	14	102	2	1	57	2	6

## Data Availability

All data are present in the article.
